# Un cas de gigantomastie gravidique bilatérale

**DOI:** 10.11604/pamj.2019.32.50.17648

**Published:** 2019-01-29

**Authors:** Adja Coumba Diallo, Mouhamadou Bachir Ba

**Affiliations:** 1 Institut Joliot Curie de l'Hôpital Aristide Le Dantec, Dakar, Sénégal

**Keywords:** Hypertrophie mammaire, sein, grossesse, mastectomie, Breast hypertrophy, breast, pregnancy, mastectomy

## Abstract

We here report the case of a 29-year old gravida 2, para 2 patient with no particular past medical history. Symptoms evolved over 2 months and were marked by bilateral breast growth impairing her daily activities. Clinical examination showed hypertrophied breasts and bilateral breast ulcers. She had a history of 28-weeks amenorrhea. Anatomopathological examination of ulcers showed fleshy bud-like tissue. The patient had high levels of prolactin (1345 µUI/ml). The levels of FSH and LH were normal. The patient underwent bromocriptine therapy without success. Patient's evolution was marked by decrease in size and regression in skin ulcers six months after vaginal birth. Gestational gigantomastia is a breast hypertrophy characterized by a breast volume exceeding 1500 cm^3^ . Its cause is unknown. Radical treatment is based on bilateral mastectomy.

## Images in medicine

Il s'agissait d'une patiente de 29 ans, sans antécédent pathologique, II gestes II pares. La symptomatologie évoluait depuis 2 mois, marquée par la survenue d'une augmentation mammaire bilatérale gênant les activités quotidiennes. L'examen physique montrait des seins hypertrophiés, des ulcérations mammaires bilatérales. Elle présentait une aménorrhée de 28 semaines. L'examen anatomopathologique des ulcérations est revenu en faveur d'un bourgeon charnu. La prolactinémie était élevée (1345 µUI/ml). Les taux de FSH et de LH étaient normaux. Un traitement à base de bromocriptine a été réalisé sans succès. L'évolution était marquée par une diminution de la taille et la régression des ulcérations cutanées six mois après l'accouchement par voie basse. La gigantomastie gravidique est une hypertrophie mammaire dont le volume dépasse 1500 cm^3^ durant la grossesse. L'étiologie n'est pas connue. Le traitement radical repose sur la mastectomie bilatérale.

**Figure 1:**
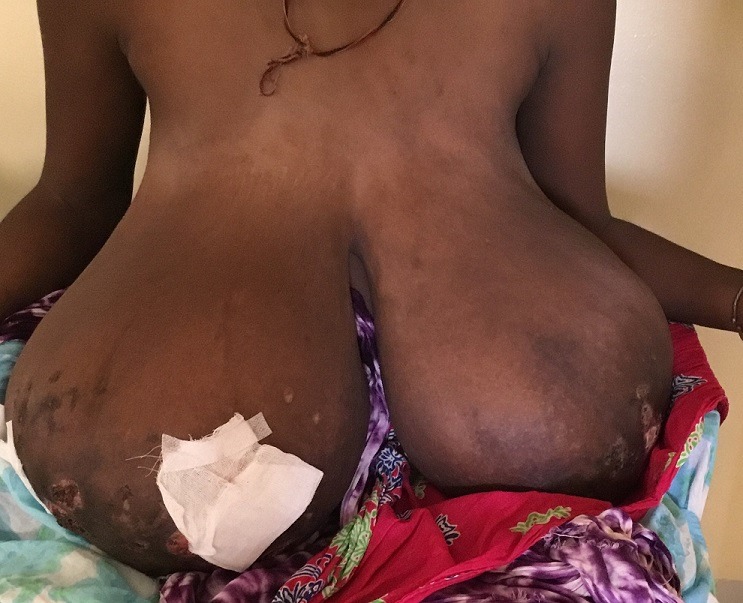
Gigantomastie bilatérale avec des ulcérations cutanées

